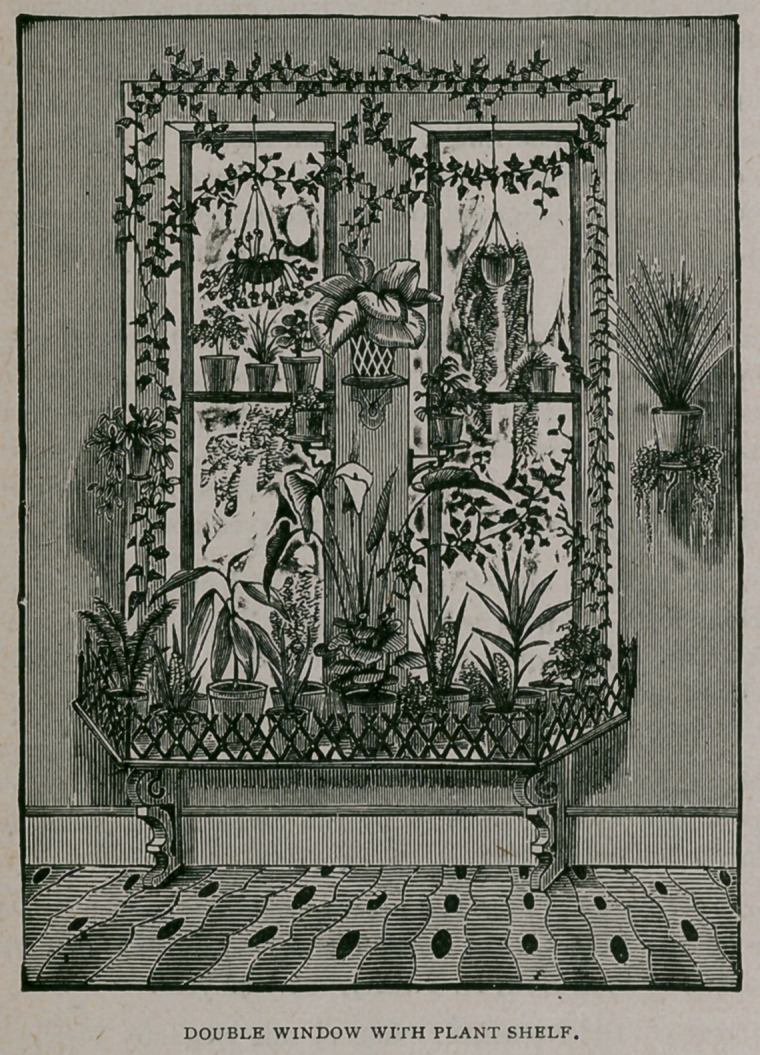# Household

**Published:** 1887-12

**Authors:** 


### HOUSEHOLD.

WINDOW GARDENING.—What adds more to the cheerfulness of the home during the lonely, dreary days of winter, than flowers ? All can have them, the poor as well as the rich, if a little care and forethought is used in growing and arranging them.

DOUBLE WINDOW WITH PLANT SHELF.

The preparatory work consists in transplanting and fairly starting in small pots, in August or September, the Madeira vine, creeping Charlie, cypress vine, balloon vine the common English, the German, or the Kenilworth ivy, or morning glory, flowering bean, or sweet-scented pea, or, if you are disposed to be more aristocratic, smilax, lophiospermum, or, if the window is large, and the foilage is not deemed too rank, the clematis or the passion vine. Nearly all of these, if thus started, will grow finely and festoon your windows in a few weeks ; some of them have fine blossoms, which will add to the beauty of their foilage. Next, for the plants to make a display in your windows. What these shall be, and how they shall be arranged, depends very much upon the size, shape and character of your windows. If you have a a bay or oriel window, either large or small, you can make it the most attractive feature of your room at a very small expense. First place your pots with climbing vines at the sides on low brackets, and the vines to make a beautiful frame for your windows. If the window is a deep bay, other and more delicate vines may be placed between the side windows and the main one—such as smilax, the Kenilworth ivy, or the cypress vine—and trained over the ceiling of the bay. At the base of the windows have a shelf six to eight inches wide (eight is best), supported by the ordinary metal brackets, and in front tack the expanding framework (such as is shown in the Fig.), which is now to be found for sale by the yard very cheap, at all the flower stores—the black walnut is the prettiest, though the holly wood is very neat ; stretch it to its full extent before tacking it on. Then selecting your hardiest and most freely-blooming plants—geraniums, pelargoniums, rose geraniums, all from slips potted in July or August, periwinkles, fuchsias, heliotropes, bouvardias, cuphias, and newly-potted slips of verbena, with such other beautiful small plants as you may find desirable—place each pot in one about three sizes larger, which is partially filled with fine earth, and the space between loosely packed with moss. Set these on your shelf, arranging them with reference to complementary colors ; put in the center where the main partition between the two divisions of the central window is, a good and shapely ardisia, which, if it has been plunged during the summer, will, by this time, be loaded with its beautiful berries, which are in November just beginning to turn to a beautiful scarlet. These berries will hang on till Jnne ; and, while the plant is of very moderate price, it has no superior as an ornamental shrub. In the corners put callas, which should have been heeled or turned over to rest, as early as July or August 1st. Their position should be partially shaded, and where they will not have too much heat; when they begin to bud, they should have a plenty of warm, almost hot, water furnished them daily. They, too, should be placed in a pot surrounded by a large pot, and the interstices filled in with moss. Across the center of the windows place other shelves with pots of smaller flowers, and, among the rest, creeping plants, such as verbenas, sweet alyssum, nemaphila lobelia, mesembryanthemum, etc., etc. On a table in the center, if you can have a neat box, zinc-lined, you can set in pots, hyacinths, amaryllis, cyclamens, iris, and the finest sorts of crocus, and, packing moss around them, keep them moist. From the ceiling of the bay may be suspended hanging baskets, taking the precaution to keep them moist. The outlay for all this is very little, and if you are ingenious you can do it all yourself.

But everybody has not bay windows, or even double windows. For these unfortunates, among whom we are sorry to be obliged to reckon ourselves, the simpler arrangment will suggest itself almost as effective. A shelf at the foot of each window supported on brackets, and, if preferred, protected by the expanding framework, will give room for four or six pots at each window, while the vines can be trained around the windows, as in the other case. A swinging bracket large enough for two pots can be attached to the outer side of the framework of each window, midway of its height, and a rustic basket attached to a hook projecting from the top of the window frame, if desired. On a table or slab between the windows a small jardiniere, containing an ardisia, or Tahiti orange, can be placed. In the selection of climbers for trimming the windows, avoid the climbing fern, which is offered so abundantly at all the flower stores. It cannot be made to live in parlors, and in spite of all the care which may be taken with it will soon become dry and unsightly. The ivies, Madeira vine and cypress vine are the best, though several other climbers are pretty. The blossoms of the Maderia vine, which will come out if it is well cared for in February or March, are very fragrant, and will fill the parlors with their delicate perfume.

The wall pockets so plenty in these days of scroll sawing, can be very easily adapted to the purpose of plant cultivation, and add greatly to the beauty of these simple decorations.

## Figures and Tables

**Figure f1:**